# The influence of viscosity of hydrogels on the spreading and migration of cells in 3D bioprinted skin cancer models

**DOI:** 10.3389/fcell.2024.1391259

**Published:** 2024-05-21

**Authors:** Lissinda H. Du Plessis, Chrisna Gouws, Daniel Nieto

**Affiliations:** ^1^ Centre of Excellence for Pharmaceutical Sciences, Faculty of Health Sciences, North-West University, Potchefstroom, South Africa; ^2^ Advanced Biofabrication for Tissue and Organ Engineering Group, Interdisciplinary Centre of Chemistry and Biology (CICA), Faculty of Health Sciences, University of Coruña, Campus de A Coruna, Coruna, Spain

**Keywords:** skin cancer, melanoma, 3D bioprinting, hydrogels, cell interaction

## Abstract

Various *in vitro* three-dimensional (3D) tissue culture models of human and diseased skin exist. Nevertheless, there is still room for the development and improvement of 3D bioprinted skin cancer models. The need for reproducible bioprinting methods, cell samples, biomaterial inks, and bioinks is becoming increasingly important. The influence of the viscosity of hydrogels on the spreading and migration of most types of cancer cells is well studied. There are however limited studies on the influence of viscosity on the spreading and migration of cells in 3D bioprinted skin cancer models. In this review, we will outline the importance of studying the various types of skin cancers by using 3D cell culture models. We will provide an overview of the advantages and disadvantages of the various 3D bioprinting technologies. We will emphasize how the viscosity of hydrogels relates to the spreading and migration of cancer cells. Lastly, we will give an overview of the specific studies on cell migration and spreading in 3D bioprinted skin cancer models.

## 1 Introduction

The most important aim of the skin cancer research field is to develop safe and effective treatments that can cure patients. To advance the field, researchers will continue to rely on improved pre-clinical *in vivo* and *in vitro* skin cancer models. These must provide an accurate representation of skin cancer development, plasticity, heterogeneity, progression, and metastasis (Rebecca et al., 2020). Numerous studies aimed to develop three-dimensional (3D) bioprinted skin models, as reviewed by Ansaf et al., 2023, however relatively few studies developed 3D bioprinted skin cancer models. The role of extracellular matrix (ECM) stiffness is well-known in epithelial cancer progression and is well-studied, as reviewed by Micalet et al., 2023. The role of hydrogels with tunable elastic moduli on the adhesion, cell spreading, migration, proliferation, apoptosis, stem cell differentiation, tumor progression, metastasis, and drug response is well known (Shen et al., 2014; Chaudhuri et al., 2020). Again, only limited studies are published on 3D bioprinted skin cancer models. A distinct advantage of 3D bioprinting is the layer-by-layer biofabrication of tissue constructs, making it ideal for skin cancer models. However, there are unique challenges related to the viscosity of the hydrogel materials during the printing process compared to after the printing process. It is therefore important to consider the printability of the hydrogel material (biomaterial inks) and the bioinks (hydrogel materials combined with cells) before, during, and after the printing process. This includes characterizing the viscosity. It is furthermore important to study the effect of viscosity in the 3 days bioprinted constructs after the printing process to determine the effect of hydrogel stiffness on cell viability, proliferation, spreading, and migration. This has been well characterized for some tumor models and 3D bioprinted skin models, but limited studies are available on 3D bioprinted skin cancer models. In this review, we will briefly outline the types of skin cancer, and available skin cancer models. We will then focus on the role of 3D bioprinting in generating skin cancer models. A special emphasis will be placed on the role of viscosity of hydrogels in the spreading and migration of cells in these 3D bioprinted skin cancer models. We will conclude with challenges and future directions that can advance this complex research field.

Skin cancer, also referred to as cutaneous cancer, is divided into melanoma and non-melanoma skin cancer, the most common of which is basal cell carcinoma and squamous cell carcinoma. Incidence rates of skin cancer vary greatly among populations and geographical locations. In addition, globally in most regions, the incidence of skin cancer is higher in males than in females (Laughter et al., 2020; Urban et al., 2021; Yang DD et al., 2023; Zhang et al., 2021). Statistics from 2019 indicated that globally for both sexes and all ages incidence of melanoma skin cancer was 0.3 million cases, squamous cell carcinoma was 2.4 million, and basal cell carcinoma was 4.0 million cases (Zhang et al., 2021). Statistics for basal cell carcinoma are usually not included in cancer registries due to the low mortality rates (Hasan et al., 2019).

**FIGURE 1:**
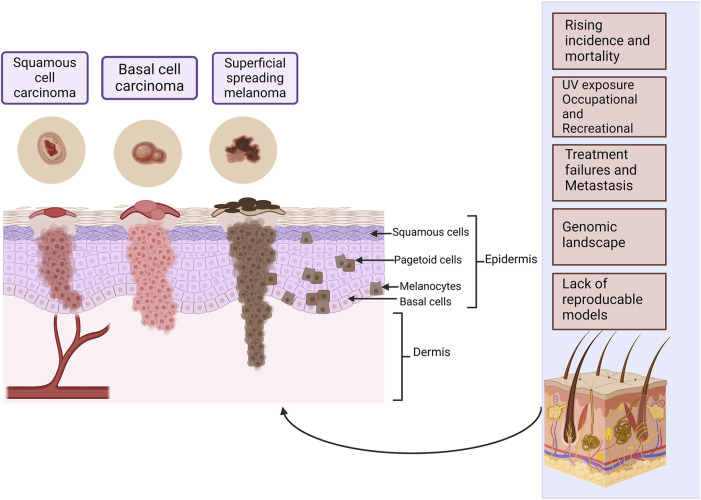
The most common types of skin cancer within the different layers of the skin. Squamous cell carcinoma arises from the squamous cells and basal cell carcinoma from the basal cell layer, these cells form the epidermal layer of the skin. Melanoma arises from melanocytes found in the basal cell layer. Melanoma has two major phases of progression. In the radial growth phase that takes place in the superficial layers of the skin, lesions are recognized as a pigmented area or plaque. In the vertical growth phase, the tumor may elevate the epidermis to give the appearance of a nodule, or it may penetrate the dermis. There may be individual abnormal melanocytes or small clusters of these cells present known as pagetoid cells or pagetoid spread of melanoma. Some of the most important challenges in skin cancer research are listed. Created with BioRender.com.

Human skin is histologically stratified into the top layer of the epidermis, followed by the dermis and the bottom layer, the hypodermis. The epidermis is further separated from top to bottom into the stratum corneum, stratum lucidum, stratum granulosum, stratum spinosum and stratum basale. The cell layers contain multiple cell types patterned in unique layers to form physiologically intact skin. Keratinocytes start in the stratum basale and migrate towards the stratum corneum in an upwards direction. Melanocytes are in the stratum basale, secreting melanin that protects against UV rays. The ECM that consists of elastic fibers, collagen, glycosaminoglycans, and proteoglycans are in the epidermis. Fibroblasts within the dermis synthesize collagen that aids in wound healing and maintaining the skin’s youthful appearance. In addition, the skin has sweat glands, hair follicles, immune cells, sebaceous glands, blood vessels, and fat deposits, each with a unique function (Powell and Soon, 2002; Agrawal and Woodfolk, 2014; Yousef et al., 2022; Ansaf et al., 2023). Skin cancer is also referred to as cutaneous cancer and frequent skin cancers include melanoma, squamous cell carcinoma, and basal cell carcinoma (Figure 1). Actinic keratoses and Bowen’s disease are also considered in discussions concerning skin cancer although they are not truly invasive tumors, because of their relationship to true skin cancers. Basal cell carcinoma and squamous cell carcinoma are non-melanoma skin cancers and they both arise from the epidermal layer of the skin (Hasan et al., 2019; Khayyati Kohnehshahri et al., 2023), whereas melanoma arises from melanocytes (Elder et al., 2020; McGovern et al., 1973). Cutaneous malignant melanoma has a low incidence rate but a high mortality rate because it is the most aggressive type of skin cancer and can metastasize rapidly, leading to a poor prognosis. The non-melanoma skin cancers are less aggressive but if they are neglected, they may grow invasively, and squamous cell carcinoma may metastasize (Hasan et al., 2019; Khayyati Kohnehshahri et al., 2023).

Malignant melanoma was historically classified in several manners, but the major categories were based on the absence or presence of the radial growth phase into superficial spreading melanoma (70%), nodular melanoma (15%), and lentigo maligna melanoma (5%). Other classifications focused on cutaneous melanoma or hair-bearing skin that is chronically exposed to the Sun and areas of the body that are not exposed to the Sun, such as the mucosa, uvea, or retina (Bernandes et al., 2021; Bobos, 2021; Dewar and Powell, 2002; Drexler et al., 2023; Elder et al., 2020; Karponis et al., 2023; McGovern et al., 1973; Schadendorf et al., 2018). The updated classification presents a multidimensional pathway approach adopted by the World Health Organization (WHO) that includes clinical, histological, epidemiological, and genetic characteristics. The main genetic drivers include B-Raf proto-oncogene (BRAF), neurofibromin 1 (NF1), and neuroblastoma RAS viral oncogene homolog (NRAS) mutations (Elder et al., 2020; Schadendorf et al., 2018). The presentation, sites of predilection, incidence, and common mutations of the different classes of melanoma are presented in Table 1.

**TABLE 1:** Classification of cutaneous malignant melanoma according to presentation, predilection sites, incidence percentage compared to all melanomas and common mutations.

Classification	Presentation	Predilection sites	Incidence	Common mutations	Ref
Melanomas developing in sun exposed skin from cumulative solar damage					
Pathway I Superficial spreading melanoma or low cumulative solar damage	Horizontal growth pattern flat or slightly elevated brown lesions with black, blue or pink discoloration which are typically greater than 6 mm in diameter and have irregular asymmetric borders	Backs of men, backs and legs of women	70% of all melanomas	Strong UV mutation signature	Drexler et al., 2023; Elder et al., 2020
				BRAF p.V600E or NRAS	
Pathway II Lentigo maligna melanoma or high continuous sun exposure melanoma	Large, irregularly shaped macules or patches with variations of tan, brown or black pigment may eventually develop a papule or nodular component	Sun-damaged skin especially the forearms and face typically occurs in the elderly	5% of all melanomas	High mutation burden with strong UV signature	Drexler et al., 2023
				NF1, BRAF^V660K^	Karponis et al., 2023
				NRAS, KIT	Elder et al. (2020)
Pathway III Desmoplastic melanoma	Spindel cell vertical growth phase with desmoplastic or rounded cell shape pattern	Areas of skin with high continuous sun exposure	1% of all melanomas	High mutation burden with strong UV signature	Drexler et al., 2023
	Firm scar like and sparsely pigmented tumor			NF1, NFKBIE, Diverse mutations in MAP kinase (Elder et al., 2020)	Karponis et al., 2023
					Elder et al. (2020)
Melanomas developing in areas protected from UV exposure or without known UV exposure					
Pathway IV Spitz melanoma	Predominantly large epithelioid cells present	Spitz nevi Most commonly in childhood	0.3%–2% of all melanomas	Frequent BRAF^V600E^ mutations	Rousi et al., 2022; Elder et al., 2020
	Large, sometimes ulcerated, history of progressive growth			HRAS	
	Nodules or papules, sparsely pigmented	Atypical Spitz tumors and Spitz melanoma more common in older adults		Fusion kinases ALK, ROS1, NTRK1, NTRK3, MET, RET, BRAF, MAP3K8	
	Well circumscribe raised borders				
	Shiny stretched epidermis				
Pathway V Acral melanoma	Aggressive form of cancer	Arise from areas protected from UV rays, including uveal or mucosal palms, soles or beneath the nail plate	8% of all melanomas	Low burden of point mutations high incidence of CCN1	Bernardes et al., 2021
	Patch lesion that enlarges radially				
	Usual ABCDE characteristics				Elder et al. (2020)
	Plaque like lesions with thick epidermis	Common type in dark complexed individuals		KIT	
	May become ulcerated or protruding nodule				
Pathway VI Mucosal melanoma	Radial growth phase with typical ABCDE features	Mucous membranes	0.8%–37% of aal melanomas	Genomic changes linked to high UV exposure	Sergi et al., 2023
	Bulky tumor that destroy surrounding tissues	Oral and nasal cavities, genital areas		KIT	Elder et al. (2020)
		Equal in all races		NRAS	
Pathway VII Melanoma arising in a congenital nevus	Three subsets	Childhood	1% of newborns	NRAS	Elder et al. (2020)
	Giant nevi covering entire body				
	Intermediate nevi				
	Small neve less than 2.5 cm				
	Similar to low-cutaneous sun exposure melanomas				
Pathway VIII Melanoma arising in blue nevus	Several categories	Both adults and children	Uncommon	G proteins GNAQ GNA11	Elder et al. (2020)
	Bulbous expansion at the base				
	Ulceration may occur	Presence of blue nevus–risk factor			
	Highly aggressive				
Nodular melanoma	Dark blue-black papule or nodule that develops rapidly	The trunk, head and neck. (This form is more frequently seen in men)	15% of all melanomas	Shares genetic changes of other melanomas	Elder et al. (2020)
		Worse prognosis than other melanomas		BRAF	
				NRAS	

## 2 Skin cancer models

**FIGURE 2:**
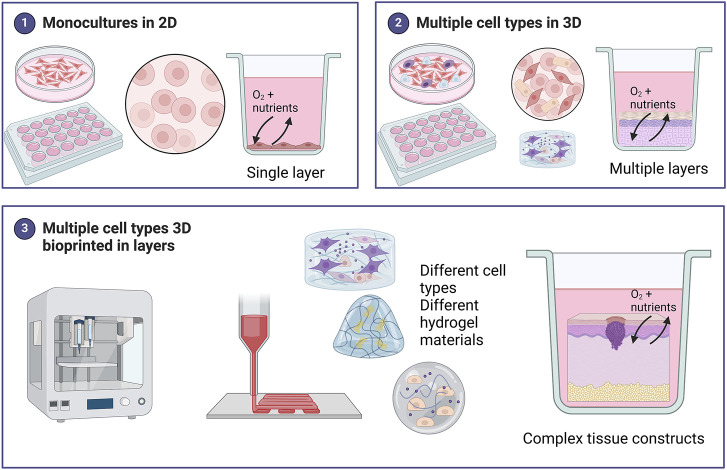
Summary of the different models used in skin cancer research. Monocultures of skin cancer cells include one cell type grown in a single layer. Various methods of 3D cell culture exist including scaffold-free techniques such as spheroids, organoids, and skin-on-chip., Scaffold-based 3D models include a hydrogel matrix. These models can be monocultures but for skin cancer, most 3D models include multiple cell types grown in layers to represent the *in vivo* skin more accurately. 3D bioprinting offers a unique advantage in being able to combine different cell types in different hydrogels (biomaterial inks) to create bioinks (hydrogel biomaterials combined with cells) for a fabricated skin model. These 3D skin cancer models can be biofabricated in a layer-by-layer approach to create complex tissue constructs more accurately. Created with BioRender.com.

Cell culture models are important tools for studying various aspects of skin cancer, and both traditional two-dimensional (2D) and newer 3D *in vitro* models allow researchers to study various complexities including cell shape, junctions, differentiation, proliferation, response to stimuli, drug responses and gene expression (Jensen and Teng, 2020). Figure 2 provides a summary of some of the different models used in skin cancer research.

Traditional 2D cell culture models of melanoma are useful for primary drug screening, molecular characterization, and invasiveness. Monocultures are free of contaminating cells, allowing for a unique understanding of changes taking place in melanocytes. More than 2000 melanoma cell lines have been developed. Established melanoma cell culture lines include MeWo, A375, SK-MEL-1, WM793, WM35, WM115, WM266-4, C32 and COLO794, and others. Despite the great diversity of melanoma cell lines, models containing BRAF or NRAS mutations are of great importance (Schadendorf et al., 2018; Sobiepanek et al., 2020). Human melanoma cell lines (A375 and 526) were found to express proteins differently from human melanocytes (FOM 78). Proteomic analysis revealed that the cell lines overexpressed six proteins compared to melanocytes. The molecular differences between the cell types can enable the development of targeted therapies (Caputo et al., 2011). However, melanoma cells cultured in 2D represent a reductionist approach, since cells proliferate much faster than *in vivo*, do not represent the *in vivo* microenvironment, and are more sensitive to drugs (Leikeim et al., 2022).

The tumor microenvironment (TME) is often studied due to its importance in how cancer arises and progresses. The skin TME consists of various cells and ECM components like collagen, fibronectin, laminin, hyaluronan, and others. The cells include tumor cells, tumor stromal cells, and immune cells (Herlyn and Shih, 1994; Nagelkerke et al., 2015; Reviewed by; Sharma et al., 2023). Whereas traditional 2D flat culture methods are standardized, various types of 3D cell culture methods have been established, including hydrogel-support-based, polymeric hard material support-based, hydrophilic glass fibers, magnetic levitation, and spheroid microplates with ultra-low attachment coatings (Jensen and Teng, 2020). Various cells of different origins are used including stem cells, commercial cell lines, and patient-derived cells (Jensen and Teng, 2020; Rebecca et al., 2020). In terms of cancer research 3D models provide more accurate representations of tumors by providing a 3D structure with varying stiffness. This can replicate *in vivo* nutrient and oxygen gradients to better simulate the hypoxic TME (Deng et al., 2022; Nascentes Melo et al., 2023). This was illustrated by studying the oxygen gradients and growth of HT1080 fibrosarcoma cells in a 3D modular culture system. Acrylated hyaluronic acid (AHA) hydrogel was used with three different degrees of viscoelasticity; soft (78 ± 16 Pa), medium (309 ± 57 Pa), and stiff (596 ± 73 Pa). Oxygen levels within the hydrogel were assessed in atmospheric (21%), hypoxic (5%), and severely hypoxic (1%) conditions. The HT1080 cells that were encapsulated within the AHA hydrogels at high densities generated nonuniform oxygen gradients, while lower cell densities resulted in more uniform oxygen gradients in the atmospheric and hypoxic environments. There were no significant differences between cell spreading and growth in the different viscosity hydrogels. The authors concluded that oxygen tension influenced cell growth more profoundly than hydrogel matrix stiffness (Shen et al., 2014).

The development of accurate 3D skin cancer models is important to advance both existing and novel treatments. 3D cell cultures are important in tumor cell biology because of their ability to replicate the *in vivo* environment (Jensen and Teng, 2020). Models are simplified representations of melanoma biology in patients. To enable a thorough understanding of molecular processes, multiple models should be used that can be translated back to the patients (Rebecca et al., 2020). Skin cancer models need to reflect the complex physical, pathological, genomic, and immunological features of the respective type of cancer as illustrated for melanoma in Table 1. Various 3D human skin as well as 3D melanoma cell culture models have been reported including 3D skin reconstructs, spheroids, organoids, and capillary network formation (Rebecca et al., 2020). Various techniques are used including air-liquid interface (ALI), casting of hydrogels, hanging drop, microfluidics, and 3D bioprinting (Reviewed by Ahn et al., 2023; Fernandes et al., 2022; He et al., 2018; Marconi et al., 2018; Michielon et al., 2022; Nomdedeu-Sancho et al., 2023; Randall et al., 2018; Rebecca et al., 2020). An explant method with mechanical separation and trypsinization was also studied. The researchers found that prolonged culture of primary melanocytes leads to spontaneous formation of spheroid-like structures. Subcultures of cells from the spheroid-like structure resulted in different fractions of melanoma cell morphologies. The typical cell doubling time ranged from 33 to 42 h, depending on the specific morphologies of the cell fractions (Ścieźyńska et al., 2021). Primary melanocytes were also cultured with different biopolymer membranes, including polyvinyl alcohol (PVA) and chitosan, which resulted in different cell viability, proliferation, and migration. This was attributed to the cell-cell interaction and cell-substrate interactions that lead to spheroid formation. Cells grown on chitosan membranes had higher cell-substrate interactions than PVA, as melanocytes in PVA resulted in spheroid formation because they could not attach to the surface. Cell migration on chitosan membranes was higher, and repigmentation was more rapid (Hsiao and Young, 2019).

Both squamous cell carcinoma and basal cell carcinoma cell models are less established. 2D cell cultures of cutaneous squamous cell carcinoma include patient-derived tumor cell cultures and commercial cell lines. Various commercial human and neck squamous cell carcinoma cell lines are available. In contrast, very few squamous cell carcinomas have been derived and established. A431 is one of the few commercially available cell lines and it has been proven to form spheroids in 3D cell culture (Hassan et al., 2019; Kumah and Bibee, 2022). Organotypic cultures in Matrigel and collagen I have also been reported (Zochke et al., 2016). *In vitro* cultures of basal cell carcinoma were successfully isolated from human patients (Brysk et al., 1992; Grando et al., 1996; Lo et al., 2010).

Due to the morphology and complexity of skin, some of the 3D skin culture techniques are better suited when compared to other tissues. The gold standard for creating 3D skin cell models is the use of ALI cultures. This facilitates epidermal differentiation of keratinocytes into corneocytes for stratum corneum differentiation. The layers of the skin are generated *in vitro* by using primary human keratinocytes and dermal fibroblasts and seeding the cells onto a collagen bed on porous inserts placed in cell culture media. The inserts can be lifted after a few days to the air-liquid interface to induce cell differentiation. After approximately 12 days of ALI culture, the resulting skin has excellent differentiation (Pruniéras et al., 1983; Carlson et al., 2008; Sharma et al., 2023). Skin carcinoma models can be grown as monocultures with ALI to simulate carcinoma *in situ*. If combined with normal keratinocytes the culture mimics early epithelial dysplasia (Germain et al., 2022).

Multicellular tumor spheroid models have been shown to have better critical physiological parameters, while also expressing melanoma markers comparable to that found in skin lesions and freshly isolated patient cells. These include CD271, HIF-1α, ABCB5, and Oct4 (Marconi et al., 2018). The 3D models also allow researchers to study drug penetration time and efficacy in solid tumors (Boucherit et al., 2020; Germain et al., 2022). Findings with spheroids and cytostatic doxorubicin have proven that it is essential to maintain the drug concentration at the tumor site for at least 2 h (Baciu et al., 2022). ERK-activity has also been found to be localized more in the growing periphery of spheroids, which is also found in patient melanoma lesions, and spheroid melanoma models have been applied to study invasion capacity, BRAF targeting, zonula occludens protein 1 (ZO-1) contribution to melanoma oncogenesis, MAPK pathway responses to MEK inhibitors, among others (Beaumont et al., 2014). Melanoma organoids or tumorospheres are spheroid cultures derived from cancer or skin stem cells and can present molecular, cellular, and histological properties, complexity, and clonality of tumors (Nascentes Melo et al., 2023). Although this approach has been applied to melanoma, it presents various limitations such as low tumor heterogeneity and lack of vasculature. Spheroid and organoid models that reflect the architecture and cellular composition of tumors are often used in immune-oncology studies, especially patient-derived organoids (PDO) models. In PDO models, tumor cell tissue is collected and cultured to obtain tumor-like organs. Although PDOs typically lack stromal and immune cells, they can be co-cultured with lymphoid tissue or peripheral-blood mononuclear cells. These models have been applied to study immune-checkpoint inhibitor therapies and cancer-infiltrating lymphocyte toxicity (Zhou et al., 2023). Commercial basal cell carcinoma cell lines include the TE 354. T cell line (Xie et al., 2022).

Novel “skin-on-a-chip” models have been developed, especially to study tumor cell migration, melanoma cell invasion, and metastasis. These models recapitulate skin layer structural organization and function in a microfluidic platform with continuous perfusion. Furthermore, these models allow the study of crosstalk with stromal and immune cells, cancer cell extravasion, melanoma cell sprouting, and biochemical and biophysical cues in tumor initiation and progression. However, these models are relatively expensive, and complex and lack organized tissue and organ formation (Li W et al., 2023b; Nascentes Melo et al., 2023).

Traditional 2D cell cultures grown in monolayers on flat surfaces do not replicate the TME in terms of complex cellular and ECM structures or interactions. Compared to the 3D environment cell morphology, polarity, pH, and cell growth and division times can be significantly different (Peng et al., 2016; Jensen and Teng, 2020). Cells grown in 2D environments are flat expand in a monolayer and are poorly differentiated (Figure 2). Fewer cell junctions form with no notable cell-to-cell communication. These cells often have unnaturally high proliferation rates. Gene and protein expression is markedly different from the *in vivo* cells. All cells in the flat layer receive the same amount of nutrients and growth factors from the medium in the plate. (Jensen and Teng, 2020). This can be an advantage for studying certain aspects, but a disadvantage in studying more complex physiological processes. 3D models offer the advantage of studying complex cellular behavior, and tumor heterogeneity and offer the possibility of personalized patient models. Cells grown in 3D environments often retain their natural shape and aggregate into spheroids or tumoroids with multiple layers. The cells are well differentiated, and cell junctions are common allowing for cell-to-cell communication. These cells have realistic proliferation rates that resemble *in vivo* cells. The core of the structure or the deeper layers remains inactive due to a lower supply of nutrients and oxygen (Jensen and Teng, 2020). However, spheroid and organoid skin models have the drawback of not producing the complex arrangement of skin structures (Sharma et al., 2023). Due to the complex organization of the skin and various complexities associated with the different types of skin cancer, 3D bioprinting with layers offers an attractive alternative to 3D cultures as illustrated in Figure 2 (Fernandes et al., 2022; Shukla et al., 2022; Viegas and Sarmento, 2024).

## 3 3D bioprinting technologies and strategies

There have been significant technological advancements in additive manufacturing and rapid prototyping has directed various 3D printing as well as 3D bioprinting technologies and strategies. Traditionally 3D printing is centered around layer-by-layer assembly of various objects using metals, concrete, hard polymers, or ceramics. These objects are usually hard with high mechanical strength and in the medical field includes implants and prosthetics. 3D bioprinting is an additive manufacturing process that can be defined as the stacking and assembling of organic materials containing living cells and other biological inks (bioinks) in spatial patterns by using a computer-aided layer-by-layer deposition approach, creating a well-arranged 3D structure. The 3D structure can be any design, but most tissue constructs use a scaffold architecture in a geometric pattern (grid with different pore sizes) (Mironov et al., 2009; O’Brein, 2011; Ozbolat and Hospodiuk, 2016; Peng et al., 2016; Dattaa et al., 2018; Ma et al., 2018). 3D bioprinting also allows for the printing of patient-specific and customized products in smaller quantities at a relatively low cost (Zadpoor and Malda, 2017; Ngo et al., 2018). The development of aqueous-based, solvent-free systems enables the direct printing of biological materials into 3D scaffolds which in combination with the ability to control external as well as the internal shape and architecture of the generated biological structures allow for a positive influence on tissue engineering and integration (Murphy and Atala, 2014; Zadpoor and Malda, 2017).

The 3D bioprinting process is based on the premise of layer-by-layer deposition on constructs. In essence, it dispenses or prints a liquid, a viscous fluid called the bioink in intricate layers to form a predesigned construct like the intended tissue (Murphy and Atala, 2014; Peng et al., 2016; Ma et al., 2018; Sabzevari et al., 2023). The bioprinting process works through the extrusion of a bioink or hydrogel biomaterial combined with human cells from various sources in a bottom-up, layer-by-layer fashion until a 3D construct is built. Once the construct has been bioprinted, it is crosslinked using the UV LED curing/polymerization system or ionic solutions, depending on the bioink’s crosslinking requirements. The crosslinking system hardens the structure allowing it to be moved without losing its structure (Mironov et al., 2009; Peng et al., 2016; Leberfinger et al., 2017; Tripathi et al., 2022).

Bioinks consist of cells, growth factors, and biomaterials that mimic the ECM. It should be clearly distinguished from biomaterial inks that usually only contain the biomaterials formulated as hydrogels. Biomaterial inks are often used in characterization studies, including determining the rheology and printability before cells are added (Leberfinger et al., 2017; Groll et al., 2018; Persaud et al., 2022; Tripathi et al., 2022; Li X et al., 2023c). Fugitive inks are temporarily printed biomaterials that liquefy and are removed to form vascular networks or other structures (Kolesky et al., 2014; Peng et al., 2016). To create bioinks to facilitate skin bioprinting keratinocytes and fibroblasts are combined with biomaterial to simulate the ECM (He et al., 2018).

Cells used in bioinks can come from various sources but primary cells from patients or animals, human or animal cell lines, and stem cells are most often used (Ma et al., 2018). Stem cells are used in various bioinks because of their ability to differentiate into multiple cell types. Embryonic, induced pluripotent, and adult stem cells are all possibilities. However, some ethical concerns and challenges with the use of stem cells remain in some countries (Lang et al., 2013; Leberfinger et al., 2017; Kirillova et al., 2020).

**FIGURE 3:**
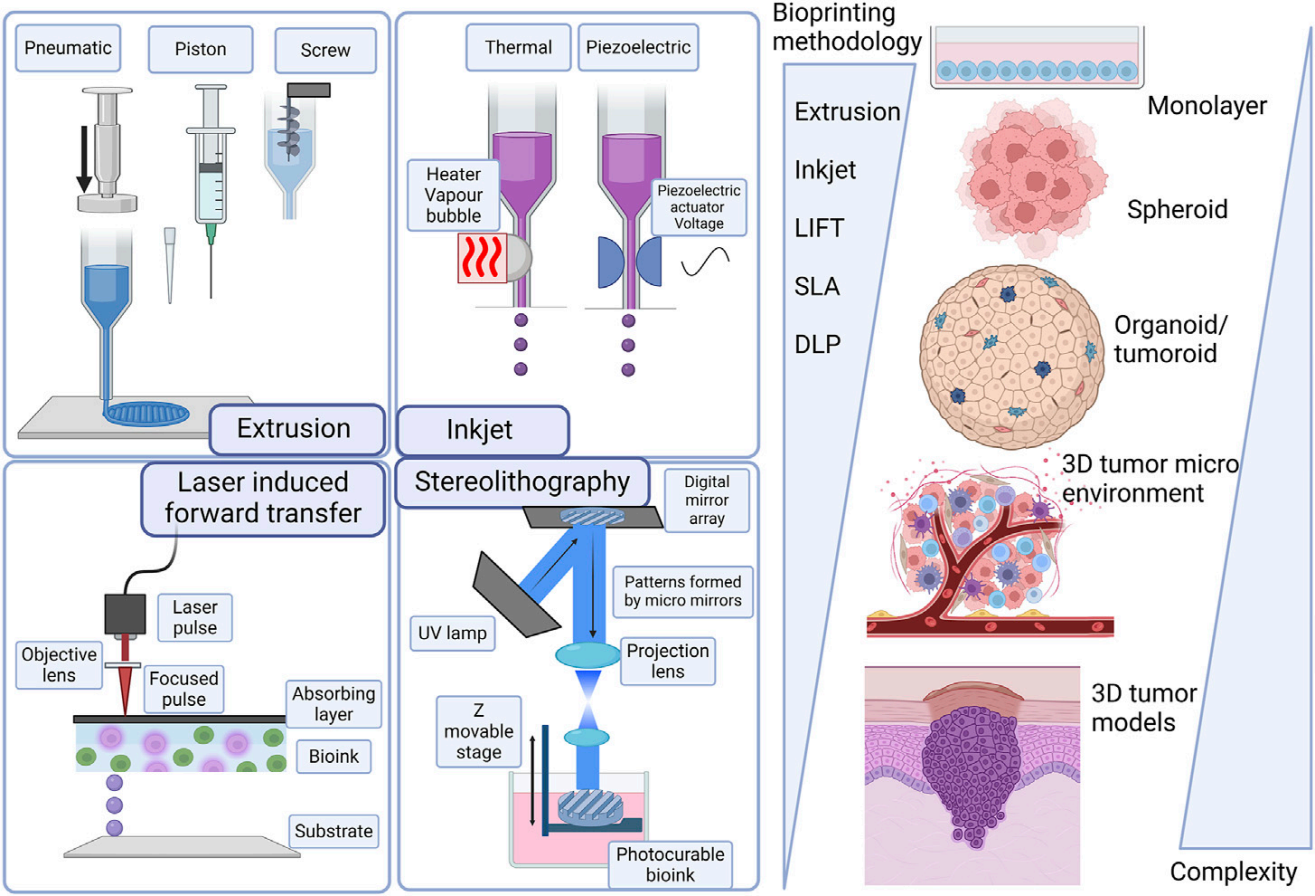
Evolution of skin cancer models and technologies from scaffold-free 3D structures to more complex 3D structures. Laser-induced forward transfer (LIFT), stereolithography (SLA), and digital light processing (DLP). Created with BioRender.com.

3D bioprinting involves various methods, materials, and equipment and has evolved over the years, giving it the ability to transform manufacturing processes (Mironov et al., 2009; Leberfinger et al., 2017; Ma et al., 2018; Ngo et al., 2018; Santoni et al., 2022; Tripahti et al., 2022). 3D bioprinting technologies can be classified under four main groups under the additive manufacturing processes proposed by the American Society for Testing and Materials (ASTM) and the International Organization for Standardization (ISO, 2020): material extrusion including extrusion-based bioprinting, material jetting including laser-assisted bioprinting and droplet-based bioprinting and vat photopolymerization including light-assisted stereolithography and digital light processing (DLP). Figure 3 summarizes the progression of skin cancer models and the importance of 3D bioprinting technologies.

Material extrusion with biopolymers has been successfully implemented in tissue engineering, and initially, this specific application was named biofabrication, micro-fabrication, or 3D bioprinting (Mironov et al., 2009; Yanagawa et al., 2016; Moroni et al., 2018). The biofabrication process encompasses the generation of tissue constructs and organs through bioprinting, bio/self-assembly and subsequent maturation (Mironov et al., 2009; Zadpoor and Malda, 2017; Ngo et al., 2018). Extrusion-based bioprinting is versatile in being able to deposit a wide array of bioinks, including hydrogels, polymer micro-and nano-carriers, tissue spheroids, cell pellets, tissue strands, and decellularized matrix components. Extrusion-based bioprinting uses different technologies to extrude hydrogel-based bioinks from the print cartridge through a needle or conical print nozzle (Leberfinger et al., 2017; Ma et al., 2018). Extrusion is facilitated mechanically either with a piston, solenoid, or screw mechanism, and pneumatically with air pressure. Pneumatic extrusion is often used, due to its range of supported viscosities of bioinks that can be used and its ability to form multilayered structures. Computer aided design (CAD) software is easily integrated into the instruments allowing continuous deposition and structural integrity of the printed constructs. This technology is hampered by slow print speeds and limited resolutions as summarized in Figure 1 (Bishop, 2017; Leberfinger et al., 2017; Ma et al., 2018; Ansaf et al., 2023).

Material jetting includes technologies where the biomaterial ink or bioink is selectively layered in the form of droplets. In droplet-based bioprinting, picolitre-sized droplets are layered on a substrate. This strategy can print low-viscosity bioink rapidly in high resolution. However, it is hampered by being able to form consistent droplets and uniformly encapsulate cells (Gudapati et al., 2016; Leberfinger et al., 2017; Sabzevari et al., 2023). Inkjet bioprinting is a form of droplet-based bioprinting that uses a hydrogel-based bioink in a print cartridge that is connected to the printer head (Figure 3). The print stage is controlled electronically and during the print process, a thermal piezoelectric actuator forms the droplets. Inkjet bioprinting includes thermal, electrohydrodynamic, electrostatic, piezoelectric, drop-on-demand, and continuous technologies. Inkjet bioprinting is low cost, with high print speeds and cell viability. A drawback of inkjet printing is that the quality of the vertical structure is poor (Singh et al., 2010; Ma et al., 2018; Sabzevari et al., 2023). Acoustic bioprinting does not use print nozzles but deposits small cell droplets with acoustic waves. In microvalve bioprinting droplets are created by using electromechanical valves. Larger droplets are created with microvalve bioprinting which leads to less resolution (Leberfinger et al., 2017).

Laser-assisted bioprinting includes laser-induced forward transfer (LIFT). With this technology a pulsing laser beam is directed towards the absorbent layer called the donor surface or ribbon (Figure 3). The energy from the laser pushes the bioink to the receiving surface. In this process a high-pressure liquid bubble forms leading to the printing of droplets. This allows for the bioprinting of structures of high resolution (Antoshin et al., 2019; Grigoryan, et al., 2021). With optimized jetting conditions, single droplets can be printed. In addition, the influence of the laser was linearly correlated with the size and volume of droplets transferred to the acceptor slide (Yusupov et al., 2020).

Light-assisted bioprinting technologies are laser-based technologies, stereolithography (SLA), and Digital light processing (DLP). Laser-based bioprinting usually provides very good resolution capabilities at high speed, but the process fails on the interaction of cells with the laser light (Antoshin et al., 2019; Grigoryan, et al., 2021) Stereolithography is a nozzle-free printing technique where a laser source, UV curing is used. It uses digital micromirror arrays to control the light intensity of each pixel for printing areas. Light-sensitive biopolymer materials are polymerized by light. Stereolithography can print light-sensitive polymer hydrogels in a layer-by-layer fashion where the printing time for each layer is similar. Stereolithography results in cell viability higher than 90% and resolution down to 200 μm (Wang et al., 2015). Digital light processing (DLP) is a bioprinting process using layer-by-layer two-dimensional (2D) crosslinking of photosensitive biomaterials when subjected to a projection with a desired pattern (Miri et al., 2019; Goodarzi et al., 2022) Table 2 provides a summary of the different 3D bioprinting technologies highlighting the viscosity of the bioink that can be used, the advantages, disadvantages and common limitations.

**TABLE 2:** Commonly used bioprinting technologies for tissue microenvironment fabrication.

Bioprinting technologies	Viscosity of bioink	Advantages	Disadvantages	Common limitations	Ref
Extrusion	30^–6^ × 10^7^ MPa s	Allows high cell density	Limited by the speed of printing for high-throughput screening	TECHNICAL	Ansaf et al. (2023)
		Low-cost technology	Moderate cell viability secondary to shear stress	NON-ACCEPTED INDUSTRIAL STANDARD SINCE 3D BIOPRINTING IS STILL EVOLVING	
		Controllable porosity	Moderate cost for high-resolution system		
		High mechanical strength			Antoshin et al. (2019)
		Prints multiple bioink simultaneously			
		Printing speed: µm/s (Microextrusion)			
		High Resolution (10–50 $\left.\mu m\right)$	Laser/cell interactions		
		Printing speed: mm/s			
Inkjet	-	Multiple reservoirs	Low speed compared to other printers	LIMITED BY THE SPEED OF PRINTING FOR HIGH-THROUGHPUT BIOPRINTING OF COMPLEX TUMOR MODELS	Gudapati et al., 2016; Sabzevari et al., 2023
		Direct incorporation of cells during printing	Non-complex architectures		
		High-throughput	Needle clogging at high ink viscosities exposing cells/high shear forces		
		No shear stress			
		Printing speed: mm/s			
Laser-based	1–300 MPa s	Viscous or solid solution	Limited scalability		Grigoryan, et al., 2021; Antoshin et al., 2019
		High Resolution (10–50 μm)	High cost		
		Printing speed: mm/s	Laser/cell interactions		
Stereolithography	No limitation	High cell viability	Monomer toxicity and use of ultraviolet radiation	BIOLOGICAL	Wang et al. (2015)
		Fast speed			
		Easy control of matrix properties	Poor hollow-structure capabilities		
		Implemented			
		Technology	Require photo-curable bioink		
		Printing speed: mm/s		FAILS TO RECONSTITUTE TISSUE-TISSUE INTERFACES AND THE TUMOUR BIOMECHANICAL ACTIVE MICROENVIRONMENT	
Digital light processing	No limitation	High printing speed	Require photo-curable bioink/typically UV in the available systems		Miri et al., 2019; Goodarzi et al., 2022
		High cell viability			
		Direct incorporation of cells during bioprinting	Customized systems/required skills		
		Dynamic bioprinting	Moderate cost for high-resolution systems/UV light can damage micromirrors	FAILS TO REPLICATE	
		High resolution			
		Printing speed: mm^3^/s			
				MULTI-SCALE TUMOUR VASCULATURE	

These light-based non-contact bioprinting tools do not present the complications related to the contact-based technologies, but the main inconvenience of UV light-based technologies emerges when cells are added to the bioink, mainly because of the toxicity of monomer and radiation (UV) can affect long-term cell viability. Nevertheless, some recent works have used visible light to polymerize the bioinks and to reduce the toxic effects associated with UV light and cell damage (Nieto et al., 2020).

In addition to the most often described technologies listed above, some alternative technologies have also been described for 3D bioprinting (Jung et al., 2022; Caceres-Alban et al., 2023). Magnetic bioprinting uses magnetic forces together with magnetic nanoparticles or magnetic microbead-labeled cells (Mironov et al., 2009). The magnetically labeled cells are organized into specific patterns using magnetic forces. The most important prerequisite of magnetic bioprinting is labeling the cells with biocompatible magnetic nanoparticles (Van de Walle et al., 2023). Other technologies include aspiration-assisted bioprinting (Ayan et al., 2020), the Kenzan method (Moldovan et al., 2017), sonolithography (Shapiro et al., 2021), and volumetric bioprinting (Bernal et al., 2019).

## 4 3D bioprinting with hydrogels

**FIGURE 4:**
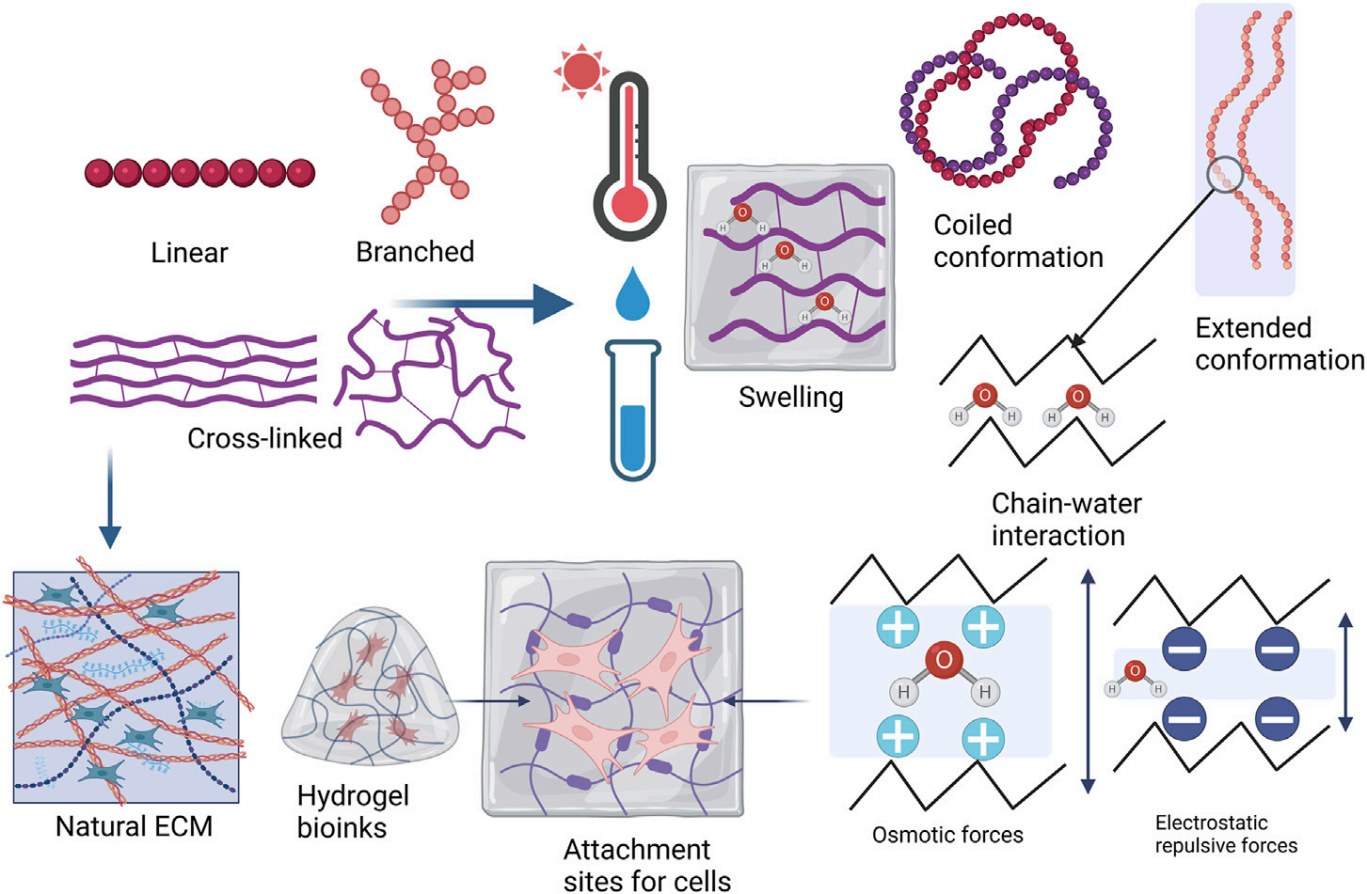
Hydrogel material polymer properties and topology, describing whether a polymer structure is linear, branched, cross-linked, or a network. Hydrogels tend to obtain freedom of movement of the polymer chains as they are placed in water or biological fluids and when temperature is increased. Swelling in a hydrogel is generally determined by polymer-solvent interaction in nonionic hydrogels and by osmotic or electrostatic repulsive forces if the hydrogel is ionic. Higher cross-linking density leads to decreased mesh size or porosity with increased stiffness. The polymer chains in the hydrogel bioink resemble the natural ECM and the swelled hydrogel provides attachment sites for cells (Adapted from Berger et al., 2004; Mehta et al., 2023; Sinko, 2011; Xu J et al., 2022a). Created with BioRender.com.

Successful 3D bioprinting relies mainly on two characteristics: cell viability and geometric accuracy. Both of these characteristics depend on the properties of the biomaterial being used and are influenced by the bioprinting parameters (Webb and Doyle, 2017). Bioinks are bioprintable materials that are based on natural and synthetic polymers (Parak et al., 2019) and are defined as cell-containing formulations that can be processed by biofabrication technology (Van Kampen et al., 2019). Biopolymers are used as the bioink during the printing process which has the necessary characteristics, including biocompatibility, biodegradability, good degradation kinetics, and safe degradation by-products and they can enhance cell migration and adhesion (or tissue biomimicry) (Cojocaru et al., 2019; Patrocinio et al., 2023). Hydrogels are often used as the basis of bioinks because of their ability to retain large amounts of water and form 3D structures (Figure 4). The polymers used in hydrogels are cross-linked, providing mechanical strength. The hydrogels are also able to swell in aqueous environments and gradually degrade over time. The typical hydrogel structure is described as a solid polymer network matrix or mesh with bound water or biological fluids. This matrix phase containing the water affords the hydrogel with elastic properties. The fluid phase imparts wetness and softness to the hydrogel, a property that enables the hydrogel to fill interstitial sites. The polymer chains resemble the natural ECM and provide attachment sites for cells. All these characteristics closely mimic natural tissues and biopolymers make hydrogels biocompatible (Ho et al., 2022; Zhou et al., 2022; Metha et al., 2023).

If one polymer cannot address the needs of the bioink characteristics or bioprinting application, its properties can be modified. Polymers may be chemically reacted or physically blended. Copolymerization is typically when more than one type of monomer is reacted randomly, alternately, as grafts or in blocks. Pluronic surfactants are typical examples of block copolymers. Interpenetrating polymer networks (IPNs) consist of two or more polymer systems. They are formed by dissolving a polymer into a solution of another type of monomer. This results in a structure where one cross-linked polymer interpenetrates into a non-cross-linked polymer system. In addition, polymers can be combined with other polymers improving the properties of the hydrogel blend (Sinko, 2011; Hospodiuk et al., 2017; Yang J et al., 2023).

Polymer topology can be described as linear, branched, or cross-linked and these affect the properties of the polymer (Figure 4). Linear polymers have chains that can freely move, but the chains also have a higher chance of approaching each other in the solid state. Branched polymers share this characteristic. The polymers may have low melting temperatures where weak intermolecular forces hold the chains together but may have higher crystallinity and melting temperatures as the chains approach each other. Chemical cross-linking restricts the polymer chains from moving freely, but the movement depends on the degree of cross-linking. Hydrogels form rigid structures with small mesh sizes and low porosity when a polymer is highly cross-linked. Increasing temperatures are generally used to process polymers and linear and branched polymers usually gain more freedom as the temperature increases. Linear and branched polymers generally dissolve in water, especially at increased temperatures, but cross-linking reduces the solubility. Cross-linking also enables the swelling behavior of hydrogels (Sinko, 2011; Ahmed, 2015; Bashir et al., 2020; Mehta et al., 2023).

In the 3D bioprinting process, the hydrogel bioinks need to have shear-thinning properties. This allows the reduced viscosity of the bioinks which allows easier extrusion through a conical nozzle or needle. The thixotropy of bioinks represents the relationship between fluid viscosity and time. This also gives an indication of stability of the hydrogels and high thixotropy indicates that hydrogels cannot be easily deformed. The ideal storage modulus (G′) compared to loss modulus (G″) measured at angular frequency indicates elastic and viscous properties. Increased G′ that is consistently higher than G″ indicates improved elastic properties over viscous properties. Determining these properties of the hydrogel bioink before bioprinting gives an indication of printability, stable physical properties, and the ability to maintain shape without collapsing after bioprinting (Das and Basu, 2019; Xu L et al., 2022b; Sánches-Sánches et al., 2023).

The printability of the bioink/hydrogel should present with structural precision and accuracy post-printing, withstand forces during the printing process, and possess characteristics that render the bioink printable. Furthermore, the bioink should be biocompatible, presenting a high cell viability post-printing, be porous enough to allow nutritional transport, and encourage cell adhesion and growth (Parak et al., 2019; Schwab et al., 2020).

After the CAD model has been printed, tissue maturation is a time-dependent process and should commence under very strict circumstances as the environment plays a distinct role in cell-cell and cell-extracellular matrix interaction (Dattaa et al., 2018). The aim of tissue engineering through 3D bioprinting is to create synthetic tissue by combining cells with scaffolds that have the relevant geometry, cues for cell growth and differentiation, enough porosity for cellular infiltration, and domains for cellular binding (Deo et al., 2020).

The TME has a complex structure consisting of cancer-associated fibroblasts, immune cells, blood, and lymphatic vessels that are suspended in the ECM. The ECM can be softer or have a stiffer structure depending on the tumor type (Malandrino et al., 2018; Deng et al., 2022). 3D bioprinting allows the prospect of laying tumor microarchitecture down to levels of 100 µm. In addition, the biomaterial inks or bioinks can be tailored to mimic the variations in ECM stiffness. The development of cancer models to study drug efficacy, drug resistance mechanisms and kinetics, TME physiology, tumor vasculature, immune cell invasion, as well as cell spreading, migration, and metastasis have all been realized with 3D bioprinted models (Reviewed by Fernandes et al., 2022; Micalet et al., 2023; Shukla et al., 2022).

## 5 Hydrogel materials used in 3D bioprinting

One of the most important considerations in biofabrication of a successful cancer model hinges on the physical and chemical characteristics of the biomaterial used as scaffold materials (Murphy and Atala, 2014). The selection of the biomaterial therefore requires an in-depth understanding of the tumor’s ECM environment (Shukla et al., 2022). A wide range of materials are used for 3D printing and may include metals, concrete, ceramics, and polymers. Generally, only polymers are suitable for mixing with cells and are the most common material used in 3D bioprinting. The polymers used are incredibly diverse and can easily be adapted to different bioprinting processes and techniques (Ngo et al., 2018). The primary polymers used during 3D bioprinting include synthetic more rigid polymers like polylactide (PLA), a polyester derived from lactic acid, and soft natural polymers including gelatin (Deo et al., 2020). Natural polymers, also called biopolymers, are polymers from living organisms, for instance, gelatin, cellulose, alginate, agarose, chitosan, hyaluronic acid, or natural gums (Li et al., 2020; Mohammadinejad et al., 2020; Li X et al., 2023b). Matrigel is the gold standard biomaterial used in 3D cell culture models. It is a solubilized basement membrane mixture able to gelate at 37°C without additional cross-linking. The main components include collagen (Type IV), laminin, entactin/nidogen, heparan sulfate proteoglycans, and several growth factors (Passaniti et al., 2022). The use of decellularized ECM to formulate hydrogels has also been used. It contains growth factors, tissue-specific signaling molecules, and the architecture native to the tissue (Ahn et al., 2017; Li X et al., 2023b).

Multi-material inks/hybrid hydrogels have slowly been replacing natural hydrogels as they provide a higher gel strength and water absorption capacity, thus they have been widely explored for solutions to the shortcomings of single-component gels (Li X et al., 2020; Maan et al., 2022; Hibbert et al., 2023). When engineering soft tissue, other biomaterials are often used in comparison to the fabrication of hard tissue. Hard tissue and soft tissue cells react differently within scaffolds as they need to undergo different cell maturation processes to obtain the final tissue construct (Rizwan et al., 2017; Van der Heide et al., 2022).

## 6 The importance of viscosity in the spreading and migration of cancer cells in vitro models

**FIGURE 5:**
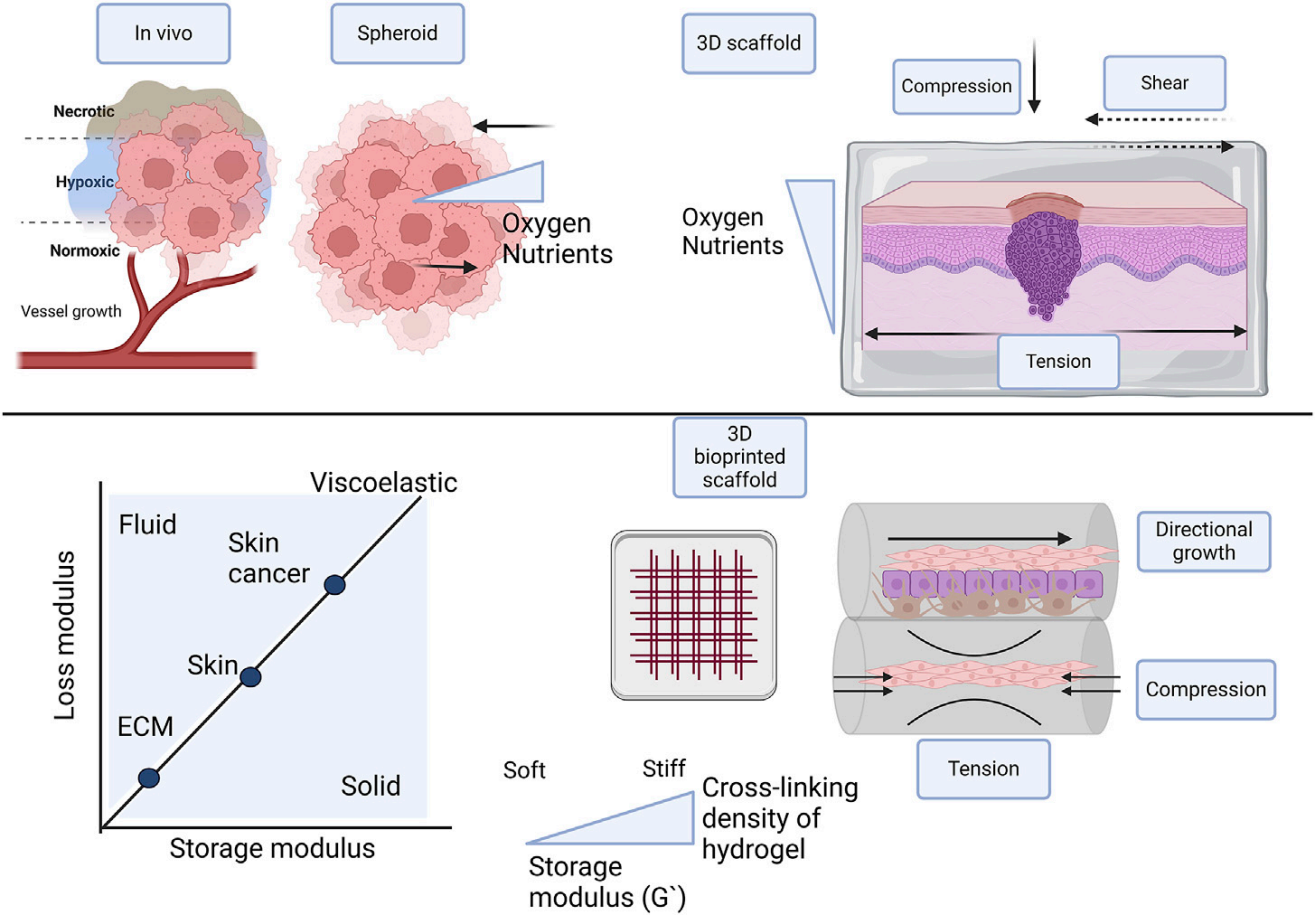
Comparison of different types of 3D skin cancer models with *in vivo* conditions, focusing on scaffold-free, scaffold-based, and 3D bioprinted scaffolds in multiple layers. To facilitate optimal growth and proliferation of cells in 3D models, oxygen and nutrient supply is important. Cell-cell interaction is influenced by mechanical forces and tissue stiffness, including compression, tension, and shear stresses. Tissue stiffness of the native ECM, skin, and skin tumor differ and should be incorporated in the skin cancer models. 3D bioprinting in layers confers directional growth. The hydrogel in the multilayer 3D bioprinted scaffold provides osmotic and hydrostatic pressure. Created with BioRender.com.

Comparable to the normal structure of the skin, various cell types make up the skin cancer tumor environment. This includes stromal cells, fibroblasts, endothelial cells, and innate and adaptive immunity cells. Stromal cells secrete ECM proteins to form a tumor-supporting environment (Herlyn and Shih, 1994; Powell and Soon, 2002; Schadendorf et al., 2018; Nagelkerke et al., 2015; Dai et al., 2018; Brás et al., 2020; Deng et al., 2022; Shukla et al., 2022). The skin TME also contains vasculature. Angiogenesis forms new blood vessels towards the hypoxic tumor center as illustrated in Figure 5. As the tumor volume increases stress on these blood vessels and abnormal signaling leads to leaky vessels (Nagy et al., 2009; Malandrino et al., 2018; Bras et al., 2020; Deng et al., 2022). The ECM in skin cancer is equally complex containing structural proteins like collagen, glycoproteins like laminin, and fibronectin; proteoglycans like decorin; ECM regulators; and secretary factors (Herlyn and Shih, 1994; Nagelkerke et al., 2015; Malandrino et al., 2018; Bhattacharjee et al., 2019; Bras et al., 2020; Deng et al., 2022). Malignant tumors containing cancer-activated fibroblasts tend to secrete collagen extensively, leading to increased stiffness. This in turn contributes to mechanotransduction signaling, EMT, migration, and metastasis. Biomaterial inks such as Matrigel and gelatin with collagen therefore closely mimic *in vivo* tumor environments (Deng et al., 2022; Shukla et al., 2022; Xiaorui et al., 2022).

In normal tumorigenesis the stiffness of the ECM or internal forces and the microenvironment or external forces plays an important role, especially in epithelial cancers (Figure 5). Stiffness is described as the way a material can deform under an applied force, measured as the elastic modulus or Young’s modulus €. Stress over strain measured in Pa or N/m^2^ are the unit of measurement and the shear G), storage (G′), and loss (G″) moduli are also measured (Baumgart, 2000). In tissue the stiffness is dictated by the ECM and the matrix density is regulated by the deposition of fibroblasts, with values of 1–10 kPa (Soumya et al., 2014; Santos et al., 2018; Ge et al., 2021). Stiffness is sensed by cells through mechanoreceptors, mainly of the integrin family and cells behave differently in varying stiffness matrices (Chen et al., 1997; Discher et al., 2005; Soumya et al., 2014). In the tumor invasion process, activated stromal cells initiate cross-linking of ECM proteins and collagen leading to altered elasticity and stiffness. This protects the cancer cells against exterior factors, including chemotherapy. The stromal cells also create tracks for the tumor cells to invade the surrounding ECM. During this invasion cells elongate, become flexible, and migrate to the vasculature to begin metastasis (Malandrino et al., 2018). Various biophysical cues including plasticity, viscoelasticity, elasticity, matrix density, the diameter of fibers, cell confinement, and alignment all contribute to the characteristics of the TME (Deng et al., 2022; Micalet et al., 2023). In spheroid factors to consider include surface tension and compression tension. Currently, there are large variations in the biomechanical properties of spheroids influenced largely by the cancer type Some studies report variations in the bulk and surface stiffness across the radius of the tumor, with a stiffer surface layer and softer core (Tian et al., 2021; Kosheleva et al., 2023). Contradicting findings report higher solid stress in the interior of the spheroid (Xin et al., 2023).

The stiffness of the skin varies according to location, age, and the layer of the skin. In addition, there are large variations in the areas measured, the techniques and the instruments used (Agache et al., 1980; Gennisson et al., 2004; Pawlaczyk et al., 2013). There are also differences between normal stiffness and skin cancer stiffness determined by the matrix stiffness of the tumor environment (Figure 5). The Young’s modulus of the epidermis (including the stratum corneum) is measured to be ∼ 4 MPa at 4–10 kHz, whereas Young’s moduli of the dermis and hypodermis are about 40 and 15 kPa, respectively, at 0.2–1 kHz (Feng et al., 2022). Normal skin stiffness has Young’s modulus values of between 1.1–210 kPa (Reviewed by Joodaki et al., 2018), whereas values reported in skin cancer were 52 ± 45 kPa (Tilleman et al., 2004).

The mechanical properties of the bulk or bioprinted hydrogel including Young’s modulus and compression modulus indicate the construct’s stiffness. Knowing these values and correlating the values with tumor tissue stiffness can help mimic the *in vivo* tumor elasticity or rigidity (Shukla et al., 2022). Another important consideration is the rheological properties that describe the flow and deformation of the hydrogel under pressure (Ferry, 1980). The rheology includes the hydrogel’s linear viscosity in response to shear strain sweep, the viscous property in response to shear rate, and the gelation kinetics (Terech and Weiss, 1997; Shukla et al., 2022; Bercea, 2023).

Methods to characterize hydrogel network formation structures and stiffness include microscopy and rheology as reviewed by Solbu et al., 2022. Microscopy is considered a direct technique and allows for the characterization of the inner, crystalline microstructure of the hydrogel. Atomic force microscopy (AFM), transmission electron microscopy (TEM), scanning electron microscopy (SEM), and optical microscopy are used. Indirect methods include rheology, cryoporositometry, low-field NMR, release tests, and dynamic light scattering. Methods to characterize cell migration and spreading rely heavily on microscopic observation and tracking cell movement with mathematical models. Studying the influence of viscosity in 2D models can prove whether cells detach and migrate from the flat surface of the culture dish. Studying the influence of viscosity in 3D models can provide information on the differences between surface and core stiffness, nutrient and oxygen supply, and the hypoxic microenvironment of the tumor (Cantini et al., 2020). A systematic review on the role of stiffness in cancer invasion studied in epithelial cancer *in vitro* models concluded that cancer invasion increases with increased stiffness of hydrogels, although some contradictory results have also been reported (Micalet et al., 2023).

## 7 The importance of viscosity on the spreading and migration of cancer cells in 3D bioprinted skin cancer models

Polymeric hydrogels are neither pure fluid nor pure elastic and are classified as viscoelastic. The deposition of hydrogel bioinks or printability through 3D bioprinting technologies depends on the shear-thinning properties of the hydrogel materials. Shear thinning increases as the viscosity increases. High viscosity improves the mechanical and structural integrity of constructs, but high hydrogel density limits cell viability and proliferation (Terech and Weiss, 1997; Fuentes-Caparrós et al., 2021; Sabzevari et al., 2023). It is therefore important to fully characterize the hydrogel or biomaterial ink (biomaterial without cells) and the bioink. This includes determining the stiffness and rheology, but also the biocompatibility, stability, swelling and erosion, and degradation kinetics. It is furthermore important to consider the viscosity of the 3D bioprinted construct on cell viability and proliferation for an extended period after the 3D bioprinting process (Shukla et al., 2022; Bercea, 2023).

3D bioprinting offers a few unique advantages for studying spreading and migration as reviewed by Shukla et al., 2022 and proved by others. There are various bioprinting strategies, and the commercial availability of both bioprinters and biomaterial inks means that researchers can easily adapt these to their needs (Santoni et al., 2022). Patterning of various bioinks that is user-defined (Schmidt et al., 2019; Maan et al., 2022) that can lead to the fabrication of complex 3D constructs with a biomimetic tumor architecture (Li, W. et al., 2023b) is possible. It allows for intricate spatial control that can enable varied cell densities (Ghose et al., 2023), or the deposition of exact positioning of spheroids into existing skin models (Moldovan et al., 2017). A wide range of biomaterials can be optimized for the specific skin tumor type, to mimic the tumor ECM stiffness and ultrastructure. Cell alignment and confinement are provided by the 3D bioprinted scaffolds, offering unique approaches to studying cell migration and spreading (Figure 5). This grants more pathophysiologically relevant models (Ruan et al., 2022; Solbu et al., 2022). Furthermore, multiple bioinks with different cell types including both tumor and stromal cells can be printed at the same time in different layers (Groll et al., 2018). There is also the possibility of integrating vascular networks into constructs (Kolesky et al., 2014). Lastly, 3D bioprinted tumor constructs allow for microenvironmental cues that induce *in vivo* like genomic and proteomic expression (Datta et al., 2020).

Both normal skin and the different melanoma models contain many layers and different cell types with specialized functions. All the layers and cell types need to be accurately presented in the model for accurate results. Therefore, replicating the complexity of fully functional skin is technically challenging (Ansaf et al., 2023; Sharma et al., 2023). The most important features of biologically equivalent skin are cellular architecture, elasticity, sensation, and mechanical strength. These properties are dependent on age, and representing the age at which a certain skin cancer is predominant is therefore important (Ansaf et al., 2023). 3D Bioprinted skin models have the potential to be used in skin regeneration and wound healing, but also to provide pre-clinical models for drug development or disease modeling (Pontigga et al., 2022; Ansaf et al., 2023; Viegas and Sarmento, 2024). There are various studies on 3D bioprinted skin models as reviewed by Ansaf et al., 2023, but there are relatively fewer studies on 3D bioprinted skin cancer models. Although the role of ECM stiffness is well-known in cancer progression and well-studied in epithelial cancers (Micalet et al., 2023), relatively few studies have been published on 3D bioprinted skin cancer models.

Some of the important findings on skin cancer cell spreading and migration in the presence of hydrogel materials came from studying non-cancerous fibroblasts. These studies did not include 3D bioprinting, focusing only on the cell-hydrogel material interactions. Cell differentiation, spreading, and migration were found to be highly dependent on matrix stiffness and the mechanical circumstances of the cells in the hydrogel. Immortalized human umbilical vein endothelial cells (HUVEC) and mouse NIH 3T3 fibroblasts were mixed with gelatin methacrylate (GelMA). The cells smoothly elongated in three GelMA percentages (5, 10% and 15% w/w). Both cell elongation and migration varied inversely with gel concentration. The study also proved that although increased hydrogel concentration may slow cell migration, it does not inhibit the process entirely (Nichol et al., 2010). In another study, NIH 3T3 fibroblasts had faster de-adhesion, cell spreading, and traction forces, in stiffer collagen-coated polyacrylamide hydrogels. The specific cell dynamics were found to be modulated by increased cell contractility (Soumya et al., 2014).

Findings on collective cell migration do not only originate from studying cancer migration and metastasis but also from wound healing (Kiran et al., 2021). Some of the latest studies also provide information on the role of the viscosity of the hydrogel on cell spreading and migration. HUVECs were used in combination with sodium alginate gellan gum and polydopamine nanoparticles, to investigate the promotion of wound healing. The mechanical strength of the hydrogel blends increased with the addition of polydopamine nanoparticles. In addition, cell migration was increased with the addition of the nanoparticles to the hydrogel. However, the cells were not 3D bioprinted but only mixed with hydrogel material. The hydrogel material was printed in wound healing scaffold with a bioplotter, and then mixed with the cells (Xu et al., 2023). Cell-directed growth was investigated using a hydrogel consisting of gelatin and microbial transglutaminase. The cell type used included mainly human skin fibroblasts, but also other cell types L929 and MC3T3. Stress was applied to the hydrogel during the printing process with a lifting motion. In addition, the hydrogel was an adhesive that allowed sustained tensile stress when the printed filament adhered to the Petri dish and was stretched. The stretching process takes place when the hydrogel is in a semi-crosslinked period. This allows for a zig-zag sewing-like process compared to traditional extrusion in *X* and *Y* directions only. After complete cross-linking, the cells appear in an ordered linear aligned pattern. The novel bioprinting method allowed for improved wound healing in mice due to multidirectional cell alignment and improved ECM secretion (Li Y et al., 2023a).

In 3D bioprinting, the choice of biomaterial ink is an important consideration not only to provide a support structure but also to facilitate cell growth and migration. Some important findings on biomaterial inks came from a comparison of melanoma cell behavior in 3D casted hydrogels, compared to 2D cells. The cell behavior of two melanoma cell lines, Mel IM and MV3, was studied in different hydrogel biomaterials. The biomaterials, alginate, alginate dialdehyde crosslinked with gelatin, and thiol-modified hyaluronan had different mechanical properties, most notably differences in stiffness. Cells grown in Matrigel and agar were used as controls. Both cell lines were able to form colonies in Matrigel and agar. The researchers also included two breast cancer cell lines in their investigation. Interestingly the melanoma cell lines had higher cell survival in denser gels, compared to the breast cancer cell lines. The authors attributed this to the Young’s modulus of normal human skin compared to cancerous skin (Schmid et al., 2020). Young’s modulus of normal human skin is 1.1 kPa–210 kPa (reviewed by Joodaki and Panzer, 2018). In cancerous skin, this value was measured as 52 ± 47 kPa (Tilleman et al., 2004), compared to 12 kPa of breast cancer tissue (Samani et al., 2003). In a follow-up study, this group focused on a comparison of melanoma cells grown in 2D and 3D alginate. In standardized 2D cell culture conditions the melanoma cell lines, Mel Wei, A375 (isolated from a primary cutaneous tumor), Mel Im, Mel Ju, and SkMel28 (isolated from malignant melanoma metastases) retained the typical spindle shape. In 0.6% alginate these cells formed dense cellular clusters after 7 days. There were significant differences in gene and microRNA expression between the cells grown in 2D compared to 3D alginate. Most notable was the downregulation of genes in RNA processing, pre-ribosomal and mitochondrial proteins, and cell cycle in 3D alginate. At the same time, processes including actin cytoskeleton organization, G protein-coupled receptor signaling, and cellular response to shear fluid stress were upregulated in 3D alginate. The gene early growth response 1 (EGR1) was the most notable upregulated gene in melanoma, with a 3-fold increase in mRNA expression compared to primary melanocytes. This highlighted the importance of EGR1 in melanoma progression (Kappelmann-Fenzl et al., 2021).

To date, the studies on 3D bioprinting of skin cancer models followed two different approaches. In the first approach the hydrogel biomaterial was extruded, or 3D printed, and the cells were added to the fabricated scaffold. In the second approach cells were mixed with the hydrogel material before 3D bioprinting. In the latter model, cells are exposed to shear stress during the bioprinting process, whereas in the first approach, this is lacking. Table 3 provides a summary of studies including 3D bioprinting of skin cancer models, with some measure of hydrogel viscosity and the influence on cell spreading and migration.

**TABLE 3:** Summary of studies highlighting the importance of viscosity importance of on the spreading and migration of cancer cells in 3D bioprinted skin cancer models. The studies are categorized into two categories: 1) studies where cells were added to already 3D printed hydrogel matrices. In these studies, the skin cancer cells were not mixed with hydrogel materials and 3D bioprinted. These cells were not exposed to the shear stress and mechanical forces of the bioprinting process; 2) Studies including the formulation and 3D bioprinting of a bioink. This includes studies where the cells are mixed with the hydrogel material and then 3D bioprinted. These cells are exposed to shear stress and mechanical stress during the print process.

Main aim	Bioink	3D bioprinting strategy	Viscosity	Spreading and migration	Viability and proliferation	References
Cells added to already 3D printed hydrogel matrices (cells not mixed with hydrogel and 3D bioprinted)						
Characterization of patient derived melanoma explants in 3D-printed collagen scaffolds	Collagen 3% (w/v)	Extrusion based with 3DX printer	ND	Microscopic observation of cells after 7 days, compared to 2D cells	MTT assay after 7 days and 21 days	Jeong et al. (2021)
	A375 cells	3D extrusion printing of collagen scaffold in frame		No specific quantitative results reported	Microscopic observation (Calcein AM and PI)	
	Patient derived melanoma explants	Adding patient derived melanoma explants to scaffold center			4-fold increase in viability in 3D collagen scaffold compared to 2D cells	
Culturing of melanoma cells in 3D printed hydrogel scaffolds and comparison with 2D cells	GelMa/PEGDA	Hydrogel scaffold 3D printed as 10 × 10 mm square scaffold with 1.2 mm height and 6 layers	Uniaxial compression testing	Microscopic observation	Microscopic observation	Duan et al. (2022)
Drug resistance	Different ratios	Extrusion with 3D bioplotter from EnvisionTEC	Youngs modulus 10–140 kPa	Cell concentrated in centers of scaffold and significant migration into hydrogels after day 7	Live/dead (calcein AM and PI)	
	A375 cells	Cells added to scaffold		Cells were confined by gel material and clustered	Number of cells counted	
					CCK-8 activity at 1, 3 5 and 7 days	
					Higher viability in 3D compared to 2D	
Melanoma and fibroblast co-culture in 3D printed hydrogel scaffolds	GelMA	Hydrogel scaffold 3D printed as 10 × 10 mm square scaffold with 1.2 mm height and 6 layers	Uniaxial compression testing	Microscopic observation	Microscopic observation	Sang et al. (2023)
Drug resistance	A375 cells	Extrusion with 3D bioplotter from EnvisionTEC	GelMA 10% and PEGDA 2.5%	Cell concentrated in centers of scaffold and significant migration into hydrogels after day 7	Live/dead (calcein AM and PI)	
	Human fibroblasts	Cells added to scaffold	Youngs modulus of 4.5–8 kP A		Number of cells counted	
				Significantly higher cell migration in co-culture compared to monoculture of A375	CCK-8 activity at 1, 3 5 and 7 days	
					Higher viability in co-culture compared to A375 monoculture	
Formulation and 3D bioprinting of bioink (cells mixed with hydrogel and then 3D bioprinted)						
Determine printability and cell behavior of two melanoma cell lines with commercially available matrices	Commercial Bioinks	Pneumatic extrusion	ND	Microscopic imaging (GFP, DsRed2) on Days 0, 7 & 14	Microscopic imaging (GFP, DsRed2) on Days 0, 7 & 14	Schmidt et al. (2019)
	Cellink	Cellink Incredible +		Even cell distribution in all layers after 14 days, no difference between gel material	Highest cell number in Matrigel	
	Cellink RGD	Grid of 1 cm^3^ with three layers		Microscopic imaging of protrusion on day 4	No proliferation in alginate hydrogel	
	GelXA			Non-significant trend between cell lines or gel material	Proliferation in clusters in Gel-MA hydrogel	
	GelXA Laminink+			Modification with RGD or laminin had no difference		
	Matrigel					
	Malignant melanoma					
	Mel IM GFP MV3dc^a^					
	1:11 cell to gel ratio					
Determine printability of alginate/hyaluronic acid/gelatin bioink for melanoma *in vitro* and study progression, tumor vascularization and metastases *in vivo*	Alginate 0.5% (w/v)	Pneumatic extrusion with Cellink Incredible +	Rheometer with plate-plate geometry at 37°C	Microscopic imaging compared to standard 2D cells; cells in hydrogel beads and to stem cells	Cell cycle analysis (FUCCI) for 7 days	Schmid et al. (2021)
	Hyaluronic acid 0.1% (w/v)	Grid of 1 cm^3^ with three layers	Storage modulus 15.5–106.9 kPa at 1 rad s^-1^	Day 14	No difference in cell cycle between day and day 7	
	Gelatin 3% (w/v)	Cross-linked with CaCl_2_		Colonies of 100 µm close to surface, often escaping gel	High cell survival and proliferation	
	Malignant melanoma Mel IM (1 × 10^6^ mL^-1^ in 3 mL gel)			No visible spreading or migration in matrix		
	ADSC					
	HEK293^b^					
Fabrication of a cancer-vascular platform with hypoxic tumor spheroids and perfusable vessel-like tubes using in suit 3D cell printing	Porcine skin derived extracellular matrix 0.5% (w/v)	Custom build with coaxial nozzles	Rheometer with plate-plate geometry at 37°C	Microscopic evaluation of spheroid formation after 48 h, compared to standard 2 D cell	Cell proliferation on day 1, 3, 5 and 7	Kim et al. (2021)
	Malignant melanoma SK-MEL-28 (10 × 10^7^ mL^-1^	Extrusion based deposition of high-density cells to form spheroids in supporting hydrogel bath creating a metastatic cancer unit	Storage modulus	Spheroids of 400, 600 and 800 µm	Delayed proliferation at 1% and 1.5% hydrogel, significantly higher proliferation in 0.5%	
	HUVECS	Incorporating vascular endothelium tubular structure	Shear thinning behavior	Hypoxic related gene expression		
	THP-1			Measurement of sprout length, increased after 48 h		
Fabrication of 3D bioprinted three-layer melanoma model containing epidermal, dermal and hypodermal layer	Agarose 3.3% (w/v)	Extrusion with REGEMAT 3D V1 bioprinter	Torsional rheometer at 25°C	Microscopic observation of maintenance of tri-layered structure	Microscopic observation	López de Andrés et al. (2023)
	Collagen Type I 1.5 mg mL^-1^	10 × 10 mm grid with height of 0.21 mm, 9 layers	Storage modulus of tri-layer 3D bioprinted construct	Mesenchymal stem cells, fibroblasts and melanoma cells	Alamar blue assay on day 1 and 14	
	Malignant melanoma A375 and Mel-1	Three different models 3D bioprinted with various layers and cell types	∼2600 kPa compared to human skin ∼2900 kPa	Comparison of A375 and Mel-1 cells	High cell viability (>90%)	
	Mesenchymal stem cells			Even cell spreading, no cluster formation	Increasing proliferation over 14 days	
	HUVECs Human keratinocytes					
	Human fibroblasts					
	Patient derived xenograft					
Embedded bioprinting of melanoma cells into microporous collagen matrices	Gelatin type A and chitosan microparticles that is used as sacrificial biomaterial ink	Custom direct ink writing	Rheometer	Microscopic observation on day 0 and 7	Microscopic observation (Acridine orange and PI)	Reynolds et al. (2023)
	Microporous collagen matrices	Embedded bioprinting	Storage modulus	Immunofluorescent staining (Ki67) proved spreading and proliferation of cells	High cell viability (>90%)	
	Murine melanoma	Use of sacrificial microparticles as rheology modifiers and microporous matrix for improved cellular activity and immune cell infiltration	Microparticles 350 Pa	Infiltration of CD8^+^ T cells 3-fold increase over 6 days	Initiation of antigen specific cell killing by cytotoxic T cells	
			Microporous collagen 30,000 Pa			
3D bioprinting of cutaneous squamous cell carcinoma model and comparison to 3D bioprinted normal skin model	Cellink SKIN	Pneumatic extrusion with BioX from Cellink	ND	Microscopic observation	Microscopic observation for 1–8 weeks	Kurzyk et al. (2024)
	Cellink Bioink	Multilayered model of 5 × 5 × 1 mm		Clear observation of three distinct layers	Live/dead staining	
	Biomaterial and cells mixed 1:1 ratio	Normal skin model with dermal fibroblasts and HaCaT cells			Immunohistochemistry	
	Primary normal human dermal fibroblasts	Squamous cell carcinoma model with dermal fibroblasts, HaCaT cells and A431 cells			Clonogenic assay	
	HaCaT cells				MTS assay	
	A431 cells				High viability and proliferation maintained for 16 weeks	

A375, Human Malignant melanoma cell line; ND, not determined; MTT, 3-(4,5-dimethylthiazol-2-yl)-2,5-diphenyl-2H-tetrazolium bromide; PI, propidium iodide; GelMA, gelatin methacrylate; PEGDA, Polyethylene (glycol) diacrylate; CCK-8, cell counting kit; RGD–Arginine, Glycine, Aspartate-peptides; XA, xanthan gum; Mel IM GFP, human melanoma cell line stably transfected with green fluorescent protein (GFP); ADSC, adipoce derived stem cells; HKE293–human embryonic kidney cells; FUCCI, fluorescent ubiquitination-based cell cycle indicator; HUVECS, Human umbilical vein endothelial cells; THP-1, human leukemia monocytic cell line; HaCat–human keratinocyte cell line; A431–squamous cell carcinoma.

^a^ MV3dc human melanoma cell line stably expressing DsRed2 and histone-2B (H2B) eGFP.

^b^ HEK293 stably expressing TNFR2-Fc-GpL.

Collagen scaffolds were used to enable the proliferation and maintenance of patient-derived melanoma explants. In the study 3D printed collagen scaffolds were fabricated with extrusion bioprinting. Cryopreserved patient-derived cells were thawed and suspended into the pre-printed collagen scaffolds. The authors found that the pre-printed collagen scaffolds accelerated the expansion of the patient-derived cells, boosted by the cell interaction in the scaffold microenvironment (Jeong et al., 2021). The drawback of this study was that the cells were not incorporated into the hydrogel material as a bioink that was 3D bioprinted. The effect of the bioprinting process could therefore not be determined.

A hybrid scaffold containing methacrylated gelatin (GelMA) and polyethylene (glycol) diacrylate was used to mimic the tumor microenvironment for A375 melanoma cells. The behavior of the cells in 3D culture was compared to cells in 2D. The scaffolds were 3D printed and the cells were added to the fabricated scaffolds. The melanoma cells aggregated within the pores of the scaffolds up until day 3 and started to migrate into the hydrogel structure on days 5 and 7. The cells grown in the scaffolds had higher levels of MMP-9 protein (a matrix metalloproteinase that remodels and degrades the extracellular matrix) compared to the 2D cells, indicating greater migration. In addition, the 3D melanoma cells were less sensitive to the drug luteolin (Duan et al., 2022). In a follow-up study by the same group the co-culture of human fibroblasts with A375 melanoma cells, using the same hydrogel and 3D printing approach was investigated. The addition of fibroblasts to the melanoma cells significantly promoted the migration of the A375 cells. The mixed cell culture was more resistant to drug treatment compared to the control (Sang et al., 2023).

Continuing from the studies on fibroblasts and melanoma cells in hydrogels, one of the first studies focusing on cell spreading and migration included two melanoma cell lines Mel Im (from melanoma metastasis) transfected with a green fluorescent protein (GFP) and MV3dc cells were 3D bioprinted with five different commercial biomaterial inks (Table 3). An extrusion-based bioprinter (Cellink Inkredible+) was used. The five different hydrogel materials included varied adhesion cues for the cells and included Cellink Bioink, Cellink RGD, GelXA, GelXA Laminink+, and Matrigel. Interestingly, the melanoma cells in all the hydrogel materials remained rounded and after 4 days single cells could be observed to develop protrusions. Although viscosity was not determined experimentally the bioprinted Matrigel constructs had the poorest printability with poor resolution scaffolds. Equal cell distribution was observed in all the bioinks, but cell viability after 14 days was the highest in Matrigel. There were significant differences between the two cell lines used with the Mel Im cell line being more sensitive to the shear forces during the bioprinting process. Gel materials containing alginate and nanofibrillar cellulose had poorer cell spreading and proliferation compared to gel materials containing gelatin methacrylate, xanthan gum, and alginate. The addition of RGD-peptide and laminin did not influence cell spreading and migration in both melanoma cell lines (Schmidt et al., 2019).

In a follow-up study by the same group, the melanoma cell line Mel Im was 3D bioprinted with an alginate/hyaluronic acid/gelatin biomaterial ink. The gel had a reported storage modulus of 15.5 kPa at 1.1 rad s^-1^ with a sixfold increase in stiffness when the alginate concentration increased by 1%. After 14 days the melanoma cells grew in large colonies close to the surface of the printed structure with the propensity to escape the gel. The authors reported this typical behavior of the melanoma cell line. The melanoma cell spreading and migration were compared to cells in 2D, melanoma cells in hydrogel beads, and stem cells to indicate differences. In addition, the role of hydrogel on melanoma behavior when vascularization is introduced was investigated. The arteriovenous (AV) loop model in immune-deficient rats was used to study proliferation, vascularization, hypoxia, immune infiltration, and metastasis over 4 weeks. The quantified ratio of tumor tissue to whole tissue excluding the remaining hydrogel was 25.9%. Microscopic evaluation of vascularization (Anti-CD31 staining) revealed vascularization in the tumor, with no positive staining observed in the hydrogel. Areas with necrosis could be observed indicating insufficient nutrient support, but CD68-positive macrophages were present in and around the tumor. Colonies of migrating melanoma clusters were observed in the surrounding tissue of the rats. Metastases were proved with positive stained HMB-45 clusters detected in the lungs of animals (Schmid et al., 2021). Although the influence of the hydrogel on *in vivo* melanoma could be described by the study, it is important to note that the *in vivo* model did not use 3D bioprinted structures, only casted hydrogel disks.

The importance of using decellularized ECM in 3D bioprinting of skin cancer was investigated using an *in situ* 3D bioprinting method. The melanoma cell line SK-MEL-28 was formulated with decellularized ECM from porcine skin. Tumors of high cellular density were directly printed as 3D spheroids within the decellularized ECM, described as *in situ* 3D cell printing of the melanoma cancer unit. In parallel *in situ* coaxial cell and polymer printing processes were used to directly incorporate a 3D vascular endothelium tubular structure. The proximity of the cancer cells relative to the vasculature promoted the epithelial-to-mesenchymal transition (Kim et al., 2021). The challenge in using material from animal sources and animal zoonoses remains a challenge in using decellularized skin from porcine skin.

The cell lines A375, Mel-1, and HUVECS were also co-cultured to establish a basic model. In addition, the researchers used mesenchymal stem cells and human fibroblasts isolated from donor skin tissue. These were embedded within an agarose-collagen type I hydrogel and 3D bioprinted with an extrusion-based bioprinter. The gel structure was maintained independent of the cell composition with the storage modulus values higher than the loss modulus values. The stiffness values of the model were comparable to human skin tissue used as control. On day 20 the viscoelastic moduli of the hydrogels changed indicating working matrix remodeling due to the cancer stem cells forming the melanoma tumor environment (Lopez-De Andres et al., 2023).

The presence of immune cells in skin cancer models and its influence on the spreading and migration of the cells is another important aspect to investigate. The role that the hydrogel porosity plays in immune cell infiltration can provide valuable insight into immune-oncology. In this model, B16-F10 melanoma cells were used as the representative cell type. A custom-built syringe system was used for bioprinting. Novel µPOROS collagen was used as sacrificial microporous biological matrices to serve as rheology modifiers and to create microporous structures when 3D bioprinted. In this study, the focus was more on an immune-based melanoma model for immune-oncology studies. The cells were combined with CD8^+^ cells and migration and infiltration of the CD8 cells were studied together with a reduction in tumor volume. The migration of the CD8^+^ cells in the µPOROS collagen was 30–40 times higher compared to GelMA or pure collagen. When combined with melanoma cells the CD8^+^ cells began infiltrating the 3D bioprinted filament within 6 h. Overall, the microporous structure facilitated cell migration, spreading, and proliferation. The antigen-specific immune cells (cytotoxic T cells) were able to efficiently migrate to the tumor site and actively mediate antigen-specific cytotoxicity (Reynolds et al., 2023). One of the limitations of this study was that human melanoma and immune cells were not used, limiting the translation to humans.

Most of the studies described focus on melanoma models with relatively fever studies on other types of skin cancer 3D bioprinted models. Kurzyk et al., 2024 describe the biofabrication of a multicellular 3D bioprinted model of human cutaneous squamous cell carcinoma. This was compared to a 3D bioprinted model representing normal skin. The response of the 3D bioprinted models to the anti-cancer drug cetuximab was also compared to 3D spheroids and cells grown in 2D. The cancer cells in the 3D bioprinted model were less sensitive to the drug compared to the spheroids and cells in 2D. Although the authors reported cell spreading, viability, and proliferation, the role of the viscosity of the hydrogels was not correlated with the findings. A previous study also reported on the development of a 3D bioprinted squamous cell carcinoma model (Browning et al., 2020). Although the results indicated the multiple layers of the 3D bioprinted model, the authors did not specifically include results on how the 3D bioprinting process and the viscosity of the hydrogel influenced cell spreading and migration.

Most of the studies highlighted in this section focused on the dynamic testing of the hydrogels or bioinks before the 3D bioprinting process. This includes rheology where sinusoidal stress or strain is applied to the biomaterial ink or bioink in a plate-plate or cone-plate geometry. The amplitude and phase shift or the response is measured to allow calculating the storage modulus (G’). The studies also included other investigations, but the storage modulus was consistently reported amongst most studies indicating the elastic behavior of the hydrogels. This was reported with the loss modulus indicating the viscous behavior (G”). Researchers use these quantitative values to inform forces required for extrusion and post-printing recovery. Of great importance is also the likely impact the printing process will have on cell viability as reviewed previously (Ning et al., 2020; Schwab et al., 2020; Cook and Rosenzweig, 2021). In most of the studies included the printability of the bioink was not specifically investigated but only inferred from theological, shape fidelity of printing constructs, and viability of cells after the printing process. Adding specific printability investigations can contribute to the standardization of 3D bioprinted models and more meaningful comparisons of data.

## 8 Conclusion

Although significant advances have been made in understanding the genomic and tumorigenesis of skin cancers, there is still a lot that we do not fully understand. The importance of 3D cell culture models is established within cancer research, but less so specifically for skin cancer. Investigations into the role of 3D bioprinting in the skin cancer field are progressing, but various challenges need to be overcome.

There are certain common identifiable challenges shared by most 3D bioprinting applications. It is a multidisciplinary field relying heavily on novel technologies. Detailed knowledge of skin cancer anatomy, pathophysiology, genomics, and clinical management is needed to be able to manufacture accurate models. Comprehensive knowledge is needed on the chemistry and rheology of polymers or biomaterial inks. In addition, specialized knowledge is needed in the mechanical engineering of novel technologies and instruments. This brings together medical specialists, healthcare workers, material scientists, biologists, chemists, mathematicians, engineers, and management to advance the field. Financial investment in human resources to create well-managed research teams has proven successful for various groups. Access to the latest novel 3D bioprinting technologies is challenging and needs economical investment from funding agencies. However, access to the latest instruments and equipment to characterize the fabricated tissues and cell models also needs investment. As cell models increase in complexity and physiological relevance, so does the need to characterize them with more advanced techniques and equipment. Long-term financial investment is therefore essential, not only for equipment but also for biomaterials. Access to biomaterials is usually less limited, but access to stem cells, patient samples, and ethics approvals can be limited depending on the country-specific guidelines. The establishment of biobanks in more recent years has lessened this burden, but it is only available in some countries and for some cancers. Finally, industrial translation and commercialization require more thought. In the strive to develop novel technologies and techniques to build increasingly complex models, scalability and cost-effectiveness are often overlooked.

In terms of 3D bioprinting of skin cancer models, challenges remain in the type of cell source chosen. There will always be high variability between different batches of cells that have been 3D bioprinted. These variations stem not only from the source but also from operators, handlers, handling techniques, culture medium batches, and the age of chemicals used. There are additional challenges in the 3D technologies used with the need for increased resolution, printing speed, reproducibility, and scaling-up. Although some studies highlighted in this review studied the physical features including tissue microarchitecture, ECM stiffness, and ECM alignment, more research is needed. There is also a continuing need to develop alternative methods to characterize the 3D bioprinted skin cancer models. This includes optical methods, but there is also a need to investigate high-throughput methods for drug screening and development. Future directions include standardization and reproducibility of models, for skin cancer models suitable biomaterials that promote tissue-specific function and maturation are needed. In terms of light-assisted 3D bioprinting systems, the development of photopolymerizable bioinks needs improvement.
